# Illuminating DNA repair in action: structural insights into a photocaged glycosylase complex

**DOI:** 10.1107/S2052252525007353

**Published:** 2025-08-28

**Authors:** Michihiro Suga

**Affiliations:** a Research Institute for Interdisciplinary Science Okayama University Okayama700-8530 Japan

**Keywords:** X-ray free-electron lasers, XFELs, time-resolved crystallography, DNA repair, hOGG1, photocaged substrate analogs

## Abstract

Structural analysis of the co-crystal between human DNA glycosylase hOGG1 and a light-sensitive substrate analog highlights its utility as a platform for real-time observation of catalytic dynamics.

Time-resolved crystallography (TRX) has emerged as a powerful technique for visualizing the dynamic behavior of biological macromolecules. In the current issue of *IUCrJ*, Imura *et al.* (2025 ▸) revisit the co-crystal structure of the human DNA glycosylase hOGG1 bound to a light-sensitive substrate analog, exploring its feasibility as a platform for capturing catalytic dynamics in real time.

Structural life science has long provided detailed snapshots of enzymes caught in various stages of their catalytic cycles. Yet these static images fall short of fully depicting the transient and dynamic nature of enzymatic function. Proteins are not rigid entities; rather, they are dynamic machines that transition through multiple short-lived conformational states, often within milliseconds. Capturing these fleeting intermediates is essential to gaining a mechanistic understanding of how enzymes function, not just how they appear at rest.

TRX is a promising approach to bridge this gap. By initiating biochemical reactions within protein crystals using external stimuli – such as light pulses or ligand diffusion – and collecting diffraction data at defined time points, researchers can construct molecular ‘movies’ of biological processes in action. This method has been especially transformative in studying light-sensitive proteins, including bacteriorhodopsin (Nango *et al.*, 2016 ▸), photoactive yellow protein (Tenboer *et al.*, 2014 ▸) and the oxygen evolving complex of photosystem II (Suga *et al.*, 2017 ▸; Li *et al.*, 2024 ▸).

However, applying TRX to enzymes that operate on chemically stable substrates – like many DNA repair enzymes – poses significant challenges. These enzymes typically lack inherent photoreactivity, making it difficult to synchronize reaction initiation within a crystalline matrix. An elegant solution lies in the use of photocaged substrates: chemically modified molecules bearing light-sensitive protecting groups that render them inert until activated by a flash of UV or visible light. This strategy offers both spatial and temporal control over reaction initiation.

In this context, Imura *et al.* revisited the co-crystal structure of human 8-oxoguanine DNA glycosylase 1 (hOGG1) bound to DNA containing a photocaged analog of 8-oxoguanine, mimicking the enzyme’s natural substrate (Imura *et al.*, 2025 ▸). hOGG1 is a central player in the base excision repair pathway, where it recognizes and excizes oxidatively damaged purines, thereby preventing mutagenic lesions from persisting in the genome. Mechanistically, hOGG1 flips the damaged base out of the DNA helix and into a catalytic pocket, where it cleaves the glycosidic bond linking the base to the sugar–phosphate backbone.

Earlier work had shown that hOGG1 could bind DNA containing a photocaged 8-oxoguanine (oG*) but could not catalyze its excision until the photolabile group was removed via illumination (Lee *et al.*, 2008 ▸). Building on this prior finding, Imura *et al.* sought to determine whether crystals of the hOgg1–photocaged-DNA complex could withstand light-induced uncaging and serve as targets for real-time structural studies.

To answer this, Imura *et al.* crystallized hOGG1 in complex with a DNA duplex containing oG*, achieving 2.81 Å resolution prior to illumination. These crystals were then exposed to 365 nm UV light to remove the photoprotecting group, activating the substrate* in crystallo* (Fig. 1 ▸). Remarkably, the illuminated crystals retained sufficient structural integrity to yield a 2.48 Å resolution structure post-photolysis.

Structural comparison revealed successful photolytic removal of the cage group, with an uncaging occupancy of 68%. The newly freed oG base was observed within the hOGG1 catalytic site. Interestingly, the enzyme did not fully adopt the active conformation associated with complete substrate excision. Instead, it assumed a late-intermediate state, partially accommodating the substrate but not achieving the whole catalytic geometry. This suggests that while the uncaging step initiated the reaction trajectory, the system may require further optimization – such as more complete uncaging or longer reaction incubation – to reach fully active states.

Nevertheless, the ability to obtain high-resolution structures before and after uncaging represents a significant technical milestone. It demonstrates that the hOGG1–oG* complex is structurally robust enough to withstand photoactivation *in crystallo*, thus enabling its use in time-resolved structural studies.

The broader impact of this work is substantial. By integrating a light-activatable DNA lesion into a crystallographically tractable system, Imura *et al.* have created a valuable platform for probing the dynamics of base excision repair. The use of photocaged DNA enables precise temporal control over reaction initiation, making it possible to collect structural snapshots at multiple, finely spaced time points.

Moreover, this approach is broadly adaptable. Other DNA glycosylases, or even unrelated DNA-processing enzymes, may be amenable to similar strategies, provided they can be co-crystallized with photoreactive substrates. The implications extend beyond academic curiosity: understanding the detailed mechanisms of DNA repair has profound relevance to aging, cancer and genome-editing technologies.

In summary, Imura *et al.* have taken a critical step toward visualizing the elusive choreography of DNA repair enzymes in real time. Their findings confirm that photocaged lesion–enzyme complexes can support structural analysis both before and after activation, offering glimpses into the conformational changes that accompany enzymatic catalysis. While further development is needed to enable true time-resolved crystallography across a complete reaction cycle, the groundwork is solidly in place.

This work exemplifies the power of chemical biology and structural ingenuity in expanding the reach of time-resolved methods. By synchronizing enzyme activity with a pulse of light, researchers are now poised to illuminate not just the static structures of DNA repair proteins, but the molecular dynamics that define how they safeguard the genome.

## Figures and Tables

**Figure 1:**
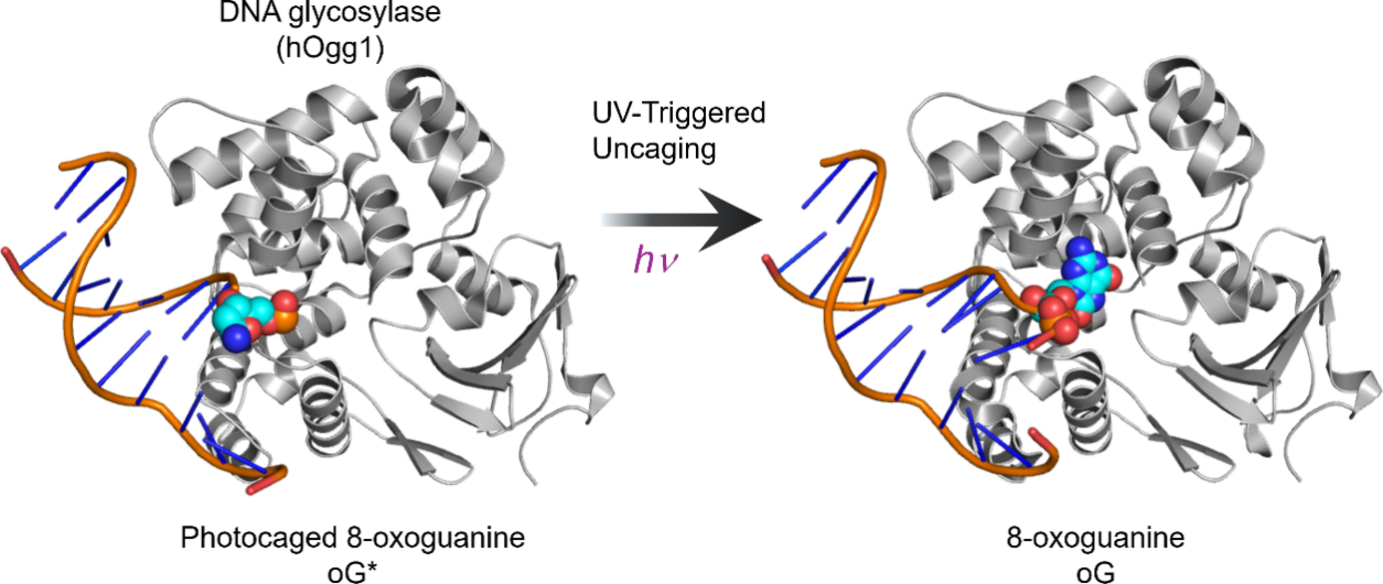
Structures of hOgg1 in complex with DNA containing photocaged 8-oxoguanine oG* (left, PDB code 9kky) and after photodissociation (right, PDB code 9kl8).
